# Application of three-dimensional fluorescence spectral characterization and chemometrics in the analysis of traceability of Paeoniae Radix Rubra

**DOI:** 10.1371/journal.pone.0328834

**Published:** 2025-08-26

**Authors:** Tong Zhou, Yao Fu, Yifan Zhang, Zhuo-Yi Meng, Hao-Dong Xu, Run Tao Tian, Chao Wang, Tian-Yu Wang, Xin-Yue Deng, Yu Zhang, LiHong Wang

**Affiliations:** 1 School of Pharmacy, Jiamusi-University, Jiamusi, China; 2 The Central Hospital of Jia Mu Si City, Jiamusi, China; 3 Chemmind Technologies (Beijing) Co. Ltd., Beijing, China; Anhui University of Chinese Medicine, CHINA

## Abstract

Natural products are treasure troves of resources that the environment has given upon humans and are directly linked to human health and well-being. Extracting natural products from medicinal plants is the material basis for treating various diseases but the natural product content of the same medicinal plant can vary due to environmental conditions, which may exert an influence on the therapeutic outcome. Since the existing identification methods for the origin of medicinal plants are cumbersome, it is necessary to find a easy, quick, and accurate way to trace the origins of medicinal plants and ensures the quality of natural products. This experiment uses chemometric techniques in conjunction with three-dimensional fluorescence technology to classify Paeoniae Radix Rubra (PRR) from various geographical sources, taking the natural products of PRR as the research object. Three-dimensional fluorescence technology can be used to identify the origin of PRR based on the presence of different endogenous luminous chemicals. In this experiment, the principal component analysis (PCA) algorithm was used to examine the overall distribution and grouping of the samples after initial characterizing the 3D fluorescence spectrum of PRR using the alternating trilinear decomposition (ATLD) algorithm. In order to predict the origin traceability of PRR samples, we combined the 3D fluorescence spectral features with four pattern recognition techniques: random forest (RF), partial least squares-discriminant analysis (PLS-DA), and k-nearest neighbor (kNN) method. The findings demonstrated that, following ATLD factorization, the sample data could successfully identify, using various models, the PRR’s production areas (Heilongjiang, Greater Khingan Mountains, Inner Mongolia, Liaoning, Hebei, Gansu, Sichuan), with 100% correct recognition rates for both the cross-validation and external validation sets. This technique offers a fresh and quick fix for PRR grading and origin tracing. Besides, this method also provides a new research idea for the origin traceability and quality evaluation of other Medicinal Plants.

## 1. Introduction

With the deepening of the research on natural products, natural products with good activity in plants have been paid more and more attention. Human beings have a long history of using plants to treat diseases, and their natural products are the main material basis for treating diseases. Soil, climate, and geographical location are the main factors affecting the content of natural products. The authentic medicinal ingredientsare cultivated in particular ecological settings and natural states. In comparison with Traditional Chinese Medicine (TCM) from other regions, their quality is higher, their curative impact is more effective, and their quality is more consistent. As a result, TCM uses them as quality benchmarks extensively. With the increasing demand for drugs in society, people often ignore the influence of regional factors on the quality of medicinal materials and plant a large number of medicinal materials at will in order to pursue economic benefits, resulting in uneven quality of TCM and difficult to control the active ingredients. When it comes to origin traceability, it is challenging to precisely track the beginnings of TCM using today’s technological tools. Consequently, establishing a quick, easy, and reliable way to track out TCM’s origins can provide quality assurance for the study of natural products of plants, and bring strong support for the research and development of natural products.

The PRR is from the dried roots of *Paeonia lactiflora* Pall. (PL) or *Paeonia veitchii* Lynch (PV). It works well to remove heat from blood and stasis in order to reduce pain. According to current study, PRR mostly consists of organic acids, tannins, volatile oils, triterpenes, flavonoids, carbohydrates, phenolic acids, steroids, and monosaccharides [1,2]. Currently, Inner Mongolia, Sichuan, Gansu, northeastern China, north China, and other regions account for the majority of the market circulation’s PRR [3]. Numerous researches on PRR have demonstrated that the chemical composition and concentration of PRR vary significantly between different locales. It is mainly affected by the following factors. PRR is the product of gene expression, which has a specific genotype and regulates the content of its elements. Cultivation, harvesting and processing activities of PRR in China are unique and affect its efficacy and safety. The third aspect is environment, including soil quality, air, water, climate, temperature and subsurface geology. Therefore, the natural environment largely determines the quality of PRR, resulting in differences in PRR from different sources. It is difficult to identify the PRR producing region by character, microscopic features, and other features when using PRR herbs, and it is nearly impossible to identify the source of PRR. Researches have employed principle component analysis (PCA) to cluster PRR from 18 production areas and radial basis function artificial neural network approach to predict PRR production areas based on the infrared fingerprints of Paeoniae Radix Rubra. According to the results, the 18 PRR samples could be loosely divided into six categories, and there was a stronger correlation between the categorization and the samples’ geographic location. In the same area, the PRR is more comparable [4]. However, this approach requires lots of time to process the samples, the instrument operating procedure is laborious, therefore, it is easily influenced by human factors, which can lead to failed predictions. Thus, the requirement to create a straightforward, quick, and precise technique for PRR geographical origin tracing is critical.

Compared to more conventional methods based on stable isotope ratio mass spectrometry (IR-MS) and other origin tracing techniques. The fluorescence approach has various benefits, including online monitoring, quick detection, high sensitivity, simple sample preparation, low cost, and easy instrument operation. A special “second-order” benefit can be obtained by combining the ATLD multidimensional calibration approach with the Excitation-Emission-Matrix (EEM) technology. In the presence of uncorrected spectral interferences, it permits the direct, quick, and simultaneous quantitative and qualitative study of multicomponent targets in complex systems. By using “mathematical separation” as an alternative to or in conjunction with “chemical and physical separation,” many qualitative and quantitative analysis goals can be achieved, improving detection efficiency and successfully lowering analysis costs at the same time. They illustrate a trend in the advancement of spectroscopic approaches toward high-dimensional analysis and significantly broaden the range of applications for fluorescence analysis methods. EEM in combination with chemometrics methods, has been successfully applied as a powerful analytical strategy for quality classification and authenticity identification of food products such as edible oils and tea leaves [5–8].

This experiment collected PRR from seven origins in China, to establish three-dimensional fluorescent fingerprints of the respective PRRs using 3D fluorescence snapshotting. Combining the alternating trilinear decomposition (ALTD) [9,10] algorithm for data processing, a novel and fast PRR origin traceability and grading scheme was established using the chemometrics technique PCA [11,12], partial least squares discriminant analysis (PLS-DA) [13,14], k-nearest neighbor method (kNN) [15], and random forests (RF) [16,17]. Using different models, the correct recognition rates reached 100% both the cross-validation set and external validation set. The established method can provide a novel and rapid solution for the origin traceability and grading of PRR, which is advantageous for preserving the integrity of medicinal flora and offers robust support for the advancement and exploitation of natural botanical products.

## 2. Materials and methods

### 2.1. Material

A total of 42 PRR samples from 7 origins were collected in October 2023, and the origin information is shown in Table 1. To eliminate the differences caused by harvesting time, strictly control the harvesting interval to not exceed 5 days. Using a random sampling algorithm, the samples of PRR from different origins were divided into a training set and a test set, and selected four predicted samples. Sample 43 was set as Predicted sample 1, which from the Langfang city in Hebei Province. Sample 44 was set as Predicted sample 2, which from the ianchang county in Liaoning Province. Sample 45 was set as Predicted sample 3, which from the Dayangshu township in Inner Mongolia Autonomous Region. Sample 46 was set as Predicted sample 4, which from the Zhang county in Gansu Province.

**Table 1:** PRR information table of different origin.

Sample	Place of origin	Serial number	Original medicinal plant	longitude	latitude
1-6	Hebei (Langfang)	hb1-hb6	PL	116.71	39.52
7-12	Heilongjiang (Dongning)	hlj1-hlj6	PL	131.12	44.06
13-18	Liaoning (Jianchang)	jc1-jc6	PL	119.83	40.82
19-24	Gansu (Zhang county)	gs1-gs6	PV	104.46	34.84
25-30	Inner Mongolia (Dayangshu)	nmg1-nmg6	PL	124.63	49.75
31-36	Sichuan (Daofu)	sc1-sc6	PV	101.12	30.98
37-42	Heilongjiang Greater Khingan Mountains (Songling)	sl1-sl6	PL	124.18	51.98

Instruments: Fluorescence spectrometer (LS55) purchased from Perkin Elmer Instruments Ltd. Analytical balance (FA2004) purchased from Shanghai Hengping Scientific Instrument Co., Ltd. CNC ultrasonic cleaner (KQ-500DE) purchased from Kunshan Ultrasonic Instrument Co., Ltd. Pulverizer purchased from Hebei Benchen Technology Co., Ltd..

Reagents: Ethanol (HPLC grade) purchased by Kaitong Chemical Reagent Co. (Tianjin. China). Ultrapure water was obtained by Milli-Q instrument (Millipore, MA, USA).

### 2.2. Methods

#### 2.2.1. Pretreatment of PRR samples.

Clean the collected PRR samples by brushing off the surface soil, removing reeds and fibrous roots, and dry them in an oven at 50 °C. After drying, crush the PRR samples and pass them through an 80-mesh sieve, place them into plastic bags, and label them for future use.

#### 2.2.2. Preparation of Sample Extracts.

First, accurately weigh 0.4 g of PRR powder, add 10 mL of 50% ethanol aqueous solution, shake well and let it stand, repeat the extraction multiple times for 2.5 hours. Ultrasonic extraction: 20 minutes, temperature: 30°C, ultrasonic power: 300 w, take 100 μL of the solution and dilute with water to 10 mL in a volumetric flask. The concentration of the sample extract is 1 mg·mL^-1^, store it sealed at 4°C for future use.

#### 2.2.3. Establishment of three-dimensional fluorescence mapping.

The sample was filtered through a 0.22 μm filter membrane, and the solution was placed in a covered cuvette, which was then placed in a fluorescence spectrometer for measurement. The relevant parameters were set as follows: Excitation wavelength (Ex): 200–375 nm (5 nm step), Emission wavelength (Em): 270–550 nm (5 nm step), and all sample data were collected using the aforementioned method. The data were then batch-imported into the ModelLab Specman3D fluorescence chemometric modeling software (Keman (Beijing) Technology Co., Ltd.) for spectral preprocessing, ATLD factor decomposition, and pattern recognition modeling analysis.

#### 2.2.4. Data analysis method.

To thoroughly and deeply distinguish the experimental data in this experiment, partial least squares-discriminant analysis (PLS-DA), k-nearest neighbor (kNN), principal component analysis (PCA) and random forest (RF) were employed.

PLS-DA was used to process the experimental data’s dimension reduction and proceeding the discriminant analysis combined with regression model. The relationship model between sample groups was established by projecting the prediction and observation variables into a new space. The discriminant threshold was used to analyze the regression results and predict the sample categories [18,19].

KNN was used to predict the sample belonging and determine the sample classification in the data set [20]. Finding K neighbors nearest to the samples to be classified by calculating the similarity between samples, and predicting the categories of the samples according to the categories of these values.

PCA was used to standardize the data, eliminate the differences between different samples, calculate the hidden associations between the data, find the main factors that affecting the samples classification, and evaluate and verify them [21–23].

RF was employed to construct the decision tree model by leveraging the data’s characteristic information [24]. The outcomes from various decision trees were aggregated to enhance both classification accuracy and stability.

## 3. Results and discussion

### 3.1. Fluorescence spectra of PRR samples

**Fig 1:**
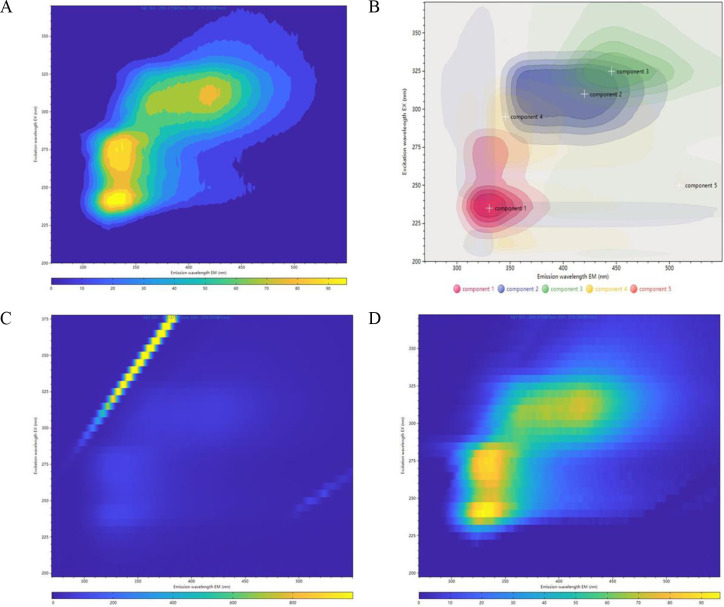
Three-dimensional fluorescence spectra of PRR samples. (A) Contour map of three-dimensional fluorescence spectra of PRR. (B) Superimposed spectra of the components of PRR after factorization. Spectra of the sample before and after deduction of 3D fluorescence background and scattering. (C) 3D fluorescence spectra before treatment. (D) Processed by blanking deduction and scattering deduction algorithms.

The raw fluorescence data of PRR were acquired by measuring each sample across excitation wavelengths of 200–600 nm and emission wavelengths of 200–650 nm. Subsequent analysis identified the characteristic fluorescence fingerprint region of PRR, demonstrating excitation and emission wavelength ranges of 200–375 nm and 270–550 nm, respectively (Fig 1a and 1b).

To address the interference of Rayleigh and Raman scattering on the trilinear structure of EEM (Excitation-Emission Matrix) spectra, piecewise spline interpolation was applied with specified bandwidth parameters: 35 nm for primary Rayleigh scattering, 5 nm for Raman scattering, and 5 nm for secondary Rayleigh scattering. Additionally, matrix data from three blank samples (50% v/v ethanol-water solution) were subtracted from each sample’s matrix data to eliminate background interference.The averaged blank sample data were subsequently used to effectively remove scattering artifacts. Representative spectra before and after this correction process are presented in Fig 1c and 1d, respectively.

**Fig 2:**
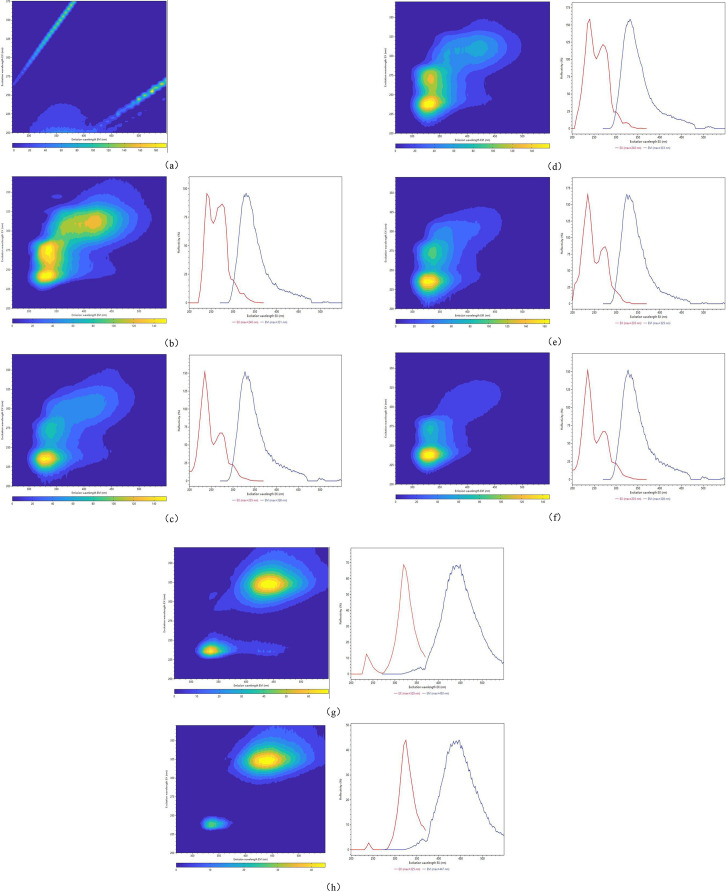
Three-dimensional fluorescence spectral contour map of PRR samples (left). Excitation and emission spectra superimposed (right). (a) Three-dimensional fluorescence spectra of methanol aqueous solution. (b-h) PRR samples (Heilongjiang, Greater Khingan Mountains, Inner Mongolia, Liaoning, Hebei, Gansu, Sichuan)..

Fig 2 showed the typical three-dimensional fluorescence fingerprints of the respective PRR (Heilongjiang, Greater Khingan Mountains, Inner Mongolia, Liaoning, Hebei, Gansu, Sichuan). Fig 2(a) presents the 3D fluorescence fingerprint of methanol, which is used for background subtraction in sample analysis. Observations from Fig 2 reveal distinct differences. For instance, in Figs 2(g) and 2(h), at the specified wavelengths (e.g., 300–350 nm for excitation and 400–500 nm for emission), the fluorescence signals from PRR samples originating from Gansu and Sichuan were more intense, whereas those from samples of other origins exhibited weaker signals. The differences in fluorescence intensity also reflected the differences in endogenous fluorescence composition and microenvironment of PRR from different origins, which provided the possibility of classification. In summary, the fluorescence characteristics, including similarities and differences in intensity, shape, and location across various or identical regions, as well as PRR fluorescence fingerprints, offer certain possibilities and challenges for subsequent classification and the construction of 3D fluorescence models.

### 3.2. ATLD algorithm-based factorization

The Alternating Trilinear Decomposition (ATLD) algorithm offers the advantage of rapid convergence, primarily due to its ability to reduce memory requirements and enhance operational efficiency. More importantly, it can accommodate an excess of group numbers, which means that the number of groups N can be greater than the target analyte. The two aforementioned advantages of ATLD make it one of the most commonly used second-order calibration algorithms.

**Fig 3:**
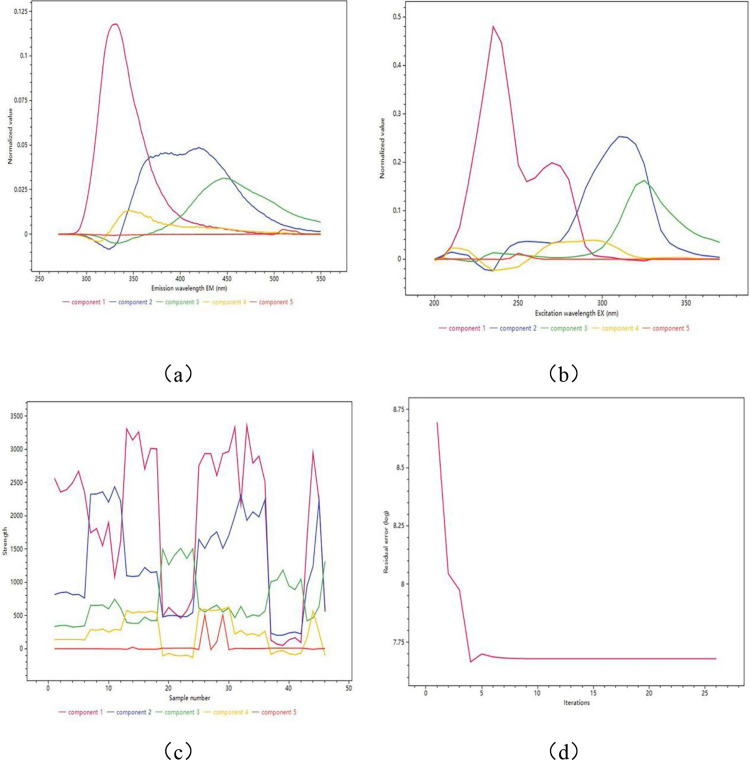
Three-dimensional fluorescence factor decomposition component spectra of the samples. (a) Excitation spectra of components (qualitative). (b) Corresponding emission spectra (qualitative). (c) The intensity distribution of different components in each sample (quantitative). (d) Factorization iterative residual plot.

First, stack the PRR sample data matrices at the same dilution level along the sample direction to obtain a three-way data array, and then apply the ATLD algorithm to the three-dimensional array. Estimate the number of components (N) using the Consistency of Replicates Consistency Diagnosis Algorithm (COR-CONDIA) before decomposing ATLD. When the CONDIA result is greater than 60%, the model can be considered to conform to trilinearity. The number of groups was finally determined to be N = 5, and the results obtained by ATLD parsing the three-dimensional array are presented in Fig 3. Fig 3(a–c) shows the excitation spectrum, emission spectrum and intensity distribution of different components in each sample respectively. When the set group score deviates from the true value, the ATLD algorithm can still yield correct element EX/EM spectra and present the excess components as background noise. Fig 3d illustrates the ultra-fast convergence of ATLD, (with results typically converging to a stable optimal solution within 10 iterations. The fluorescence data and component content differ among various origins of PRR, providing a basis for its origin tracing.

### 3.3. Multivariate statistical analysis of PRR in different producing areas

#### 3.3.1. Principal component analysis (PCA).

**Fig 4:**
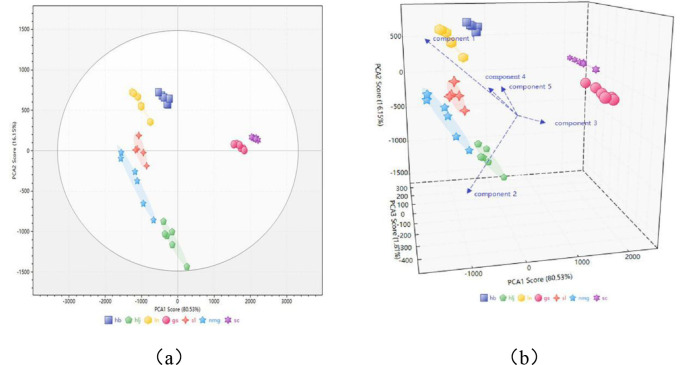
Principal component analysis score-loading plot. (a) Paepniae Radix of different origins PCA two Dimensional figure. (b) Paepniae Radix of different origins PCA Three-Dimensional figure.

**Fig 5:**
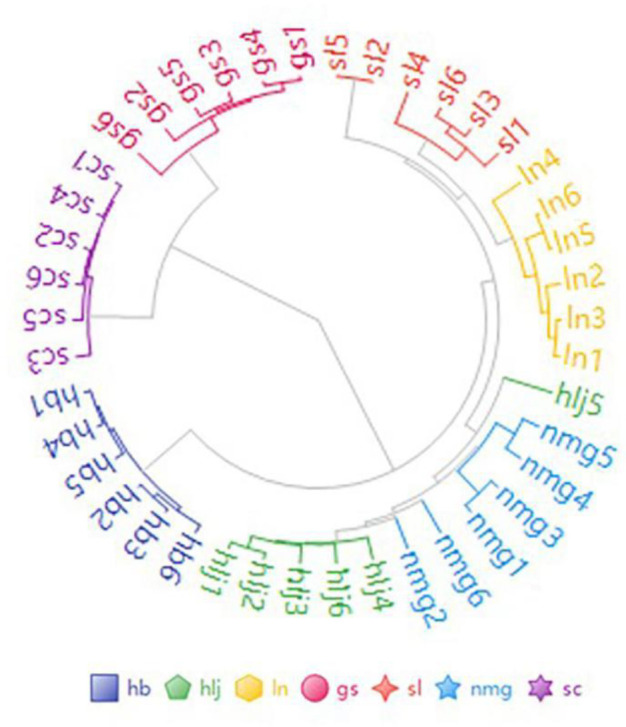
Sectoral clustering of three-dimensional fluorescence characterization factors of PRR samples from different origins.

First, the data from samples originating from various production regions of PRR were categorized into 7 groups based on their regions of origin. Use Principal Component Analysis (PCA) algorithm to analyze the differences among cimicifuga samples from different origins. The first four principal components explain more than 99.6% of the total variance of the independent variables. The results of the principal component analysis are shown in Fig 4. As an unsupervised dimensionality reduction method, the results indicate that the three-dimensional fluorescence differences among the seven types of samples are highly significant, enabling effective differentiation. Meanwhile, it can be further observed from Fig 5 that the samples from the two PV origins (Sichuan and Gansu), are significantly different from those of the other five PRR origins.

#### 3.3.2. Unsupervised cluster analysis.

Unsupervised cluster analysis is constructed by ordering simultaneously rows and columns of a data matrix by hierarchical clustering or another seriation method [25]. The analysis of the overall distribution and clustering of the samples yielded a pie cluster chart, as depicted in Fig 5. The results indicate that PRR samples from the same source can cluster together, and the three-dimensional fluorescence differences between PRR samples from different sources are significant, allowing for better differentiation.

### 3.4. Analysis of classification models

#### 3.4.1. k Nearest Neighbor (kNN) method.

**Fig 6:**
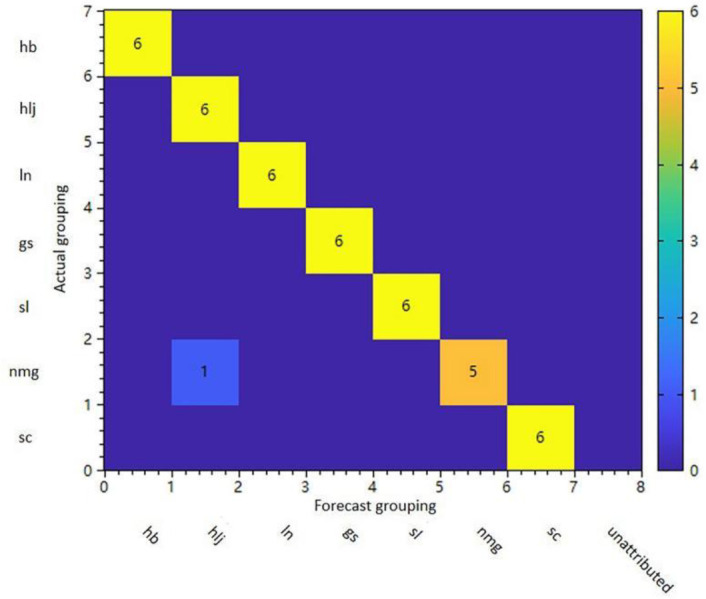
Confusion matrix of model prediction results.

Utilize the k-nearest neighbor model to classify prediction samples 1, 2, 3, and 4. The outcomes of the model identification test set predictions are presented in Table 2, and the confusion matrix for the model’s prediction results is depicted in Fig 6, with the achieved recognition rate being 100%.

**Table 2:** Model identification test set prediction grouping.

ID	Sample name	Predictive classification	Adjacent sample 1		Adjacent sample 2		Adjacent sample 3		Adjacent sample 4		Adjacent sample 5		Adjacent sample 6		Adjacent sample 7	
			Sample name	Distance	Sample name	Distance	Sample name	Distance	Sample name	Distance	Sample name	Distance	Sample name	Distance	Sample name	Distance
43	#1	hb	hb2	0.0014	hb6	0.0014	hb3	0.0014	hb4	0.0012	hb1	0.0011	hb5	0.0010	ln4	0.0009
44	#2	ln	ln6	0.0089	ln5	0.0083	ln4	0.0040	ln2	0.0038	ln3	0.0028	ln1	0.0026	sl3	0.0022
45	#3	nmg	nmg2	0.0105	nmg6	0.0034	nmg4	0.0030	hlj2	0.0023	Hlj1	0.0020	nmg4	0.0017	hlj6	0.0016
46	#4	gs	gs5	0.0088	gs2	0.0082	gs3	0.0074	gs1	0.0045	gs4	0.0041	gs6	0.0034	sc1	0.0016

#### 3.4.2. Random Forest (RF).

**Fig 7:**
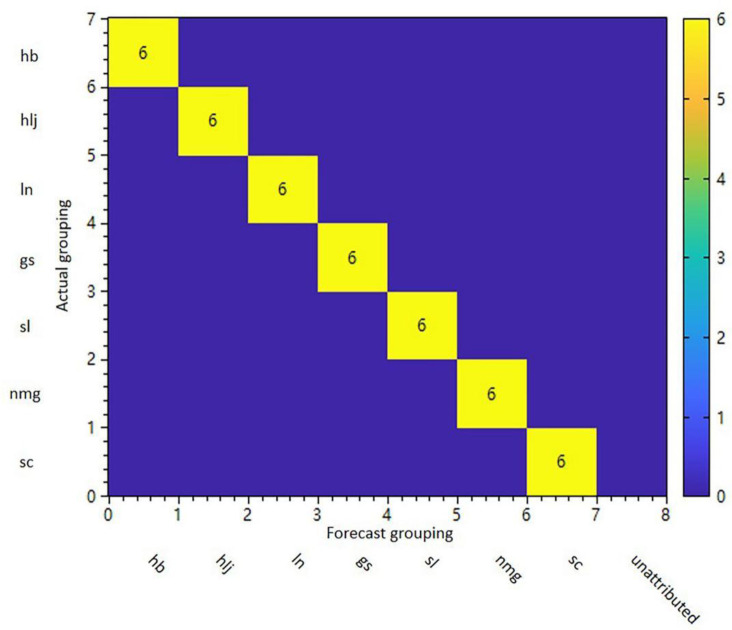
Sample pattern recognition cross-validation set confusion matrix.

The Pattern Recognition Confusion Matrix indicates that the pattern recognition model is a statistical measure of the accuracy of predictions for each class of samples, obtained through cross-validation methods. Samples located on the diagonal of the matrix in the figure represent correctly predicted samples, whereas samples that are misclassified or unattributable by the model will appear off-diagonally. These off-diagonal samples can be used to visualize the specific statistics of correctness, rejection (false negatives), and misclassification (false positives) for individual classifications. The pattern recognition statistics were shown in Table 3. Specificity and sensitivity are two quality factor parameters. Specificity indicates the model’s ability to correctly reject samples misclassified as other categories, while sensitivity denotes the model’s ability to accurately recognize samples of that category.The results indicate that the model effectively identifies this type of sample and excludes others. Using the Random Forest (RF) algorithm to predict different subgroups, the results are shown in Fig 7. Random Forest, as a nonlinear and weakly separated clustering method, has better predictive performance. The results indicate that the model’s classification accuracy for the 7 categories is 100%.

**Table 3:** Statistics of random forest model pattern recognition results of samples.

Case	Group name	Total number of samples	Training	Cross verification set	External verification set	Cross verification set			
						Reject rate%	False recognition rate%	sensibility%	specificity%
1	hb	6	6	6	0	0	0	100.000	100.000
2	hlj	6	6	6	0	0	0	100.000	100.000
3	ln	6	6	6	0	0	0	100.000	100.000
4	gs	6	6	6	0	0	0	100.000	100.000
5	sl	6	6	6	0	0	0	100.000	100.000
6	nmg	6	6	6	0	0	0	100.000	100.000
7	sc	6	6	6	0	0	0	100.000	100.000
	total	42	42	42	0	0	0	100.000	100.000

**Fig 8:**
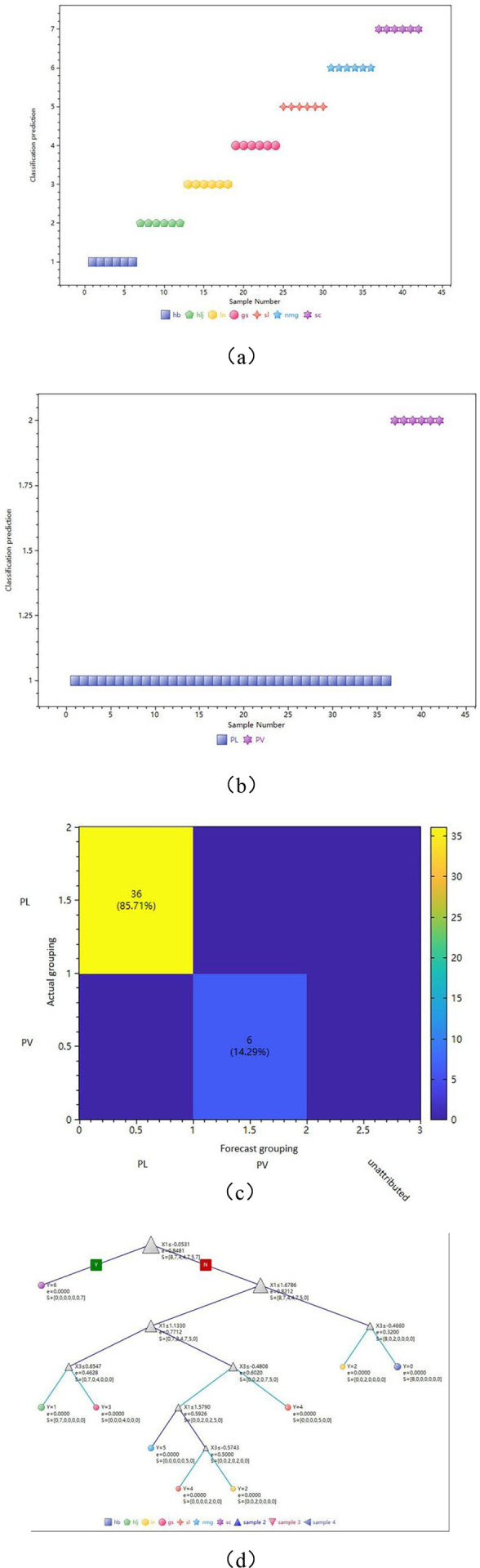
Prediction of samples from different botanical origins of PL and PV. (a) PL from different origins samples of model pattern recognition cross-validation results. (b) PL and PV samples’ model pattern recognition cross-validation results. (c) Confusion matrix for model prediction results. (d) Decision tree topology diagram schematic flow.

A random forest model was used to model the grouping of samples. The results are presented in Table 4 and Fig 8(a–c), illustrating the accuracy of the group prediction results for each sample by the two pattern recognition models.When all predictions are accurate, all samples will be positioned on the matrix’s diagonal in the figure. Conversely, if there is a discrepancy between the prediction and the actual grouping, or if there is no attribution, the corresponding number of misclassified samples will appear off the diagonal. Based on the results, it can be seen that the recognition rate is 100%.

**Table 4:** Statistics of model identification results.

Case	Group name	Total number of samples	Training	Cross verification set	External verification set	Cross verification set			
						Reject rate%	False recognition rate%	sensibility%	specificity%
1	PL	36	36	36	0	0	0	100.000	100.000
2	PV	6	6	6	0	0	0	100.000	100.000
	Sum total	42	42	42	0	0	0	100.000	100.000

Fig 8(d) illustrates the decision topology of a decision tree, serving as an example of the decision-making process. Each key variable is classified in turn using the binary tree method (≤ comparison), and ultimately correct predictions for each category of samples are obtained (marked with different colors for classification).

All independent variables (compounds) are ranked from high to low according to their contribution to the prediction grouping of the random forest (increase in binary tree purity, decrease in entropy). The results indicate that the main contributions, from high to low, are Component 1 > Component 2 > Component 5 > Component 4 > Component 3.

### 3.5. Multivariate statistical analysis of PRR in different species

#### 3.5.1. Partial Least Squares Discriminant Analysis (PLS-DA).

**Fig 9:**
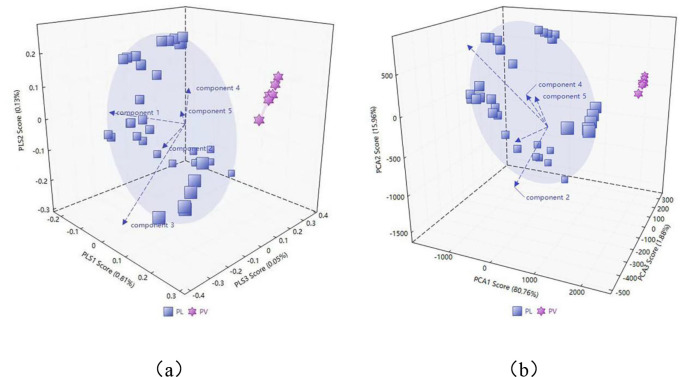
PL and PV PLS-DA pattern recognition score plots. (a) Three-Dimensional figure. PL and PV PCA pattern recognition score plots. (b) Three-Dimensional figure.

Classification of PRR using partial least squares discriminant analysis model was conducted, and the results are shown in Fig 9. From the figure, it can be seen that the PRR samples from different origins are generally divided into regions, with significant differences between those from Sichuan (sc) and Gansu (gs) compared to those from Heilongjiang (hlj), Greater Khingan Range (sl), Liaoning (ln), and Hebei (hb). The PLS-DA pattern recognition score plot indicates that different types of compounds have been well differentiated, and the results are relatively stable. The optimal number of latent variables (LVs) for PLS-DA was initially optimized through cross-validation. Based on the results of cross-validation, the LVs of PLS-DA are finally determined to be 4. In order to better evaluate the classification performance, two quality factor parameters are given: specificity, which indicates the model’s ability to reject samples from other categories, and sensitivity, which indicates the model’s ability to correctly identify samples from the target category.The specificity and sensitivity achieved by PLS-DA for each category during cross-validation range from 94.4% to 100%, indicating that PLS-DA can effectively identify samples within this category and exclude those from other categories.

#### 3.5.2. Principal component analysis (PCA).

First, divide the PRR sample data into two groups by region, separating the different categories. The Principal Component Analysis (PCA) algorithm was used to analyze the differences between the samples of PRR from different production areas.The first three principal components account for more than 98.6% of the total variance in the independent variables.The results of principal component analysis are shown in Fig 9, and the three-dimensional fluorescence differences between the two samples are significant, which enable a clear distinction.

## 4. Conclusion

Natural products play a pivotal role as a source for medicinal research and development, and stringent quality standards are essential. Three-dimensional fluorescence spectral characterization can accurately identify the fluorescent components in natural products and achieve the purpose of origin traceability through chemometric analysis. For the first time, this study introduces a detailed systematic four-step strategy of PRR for distinguishing different geographical sources. The steps include sample collection, EEM acquisition, ATLD factor decomposition, and geographical source tracking using machine learning.In the initial step, samples from seven sources were collected for PRR. The subsequent step involves extracting the sample to create a three-dimensional fluorescent fingerprint. The third step consists of decomposing the ATLD factor on the sample. Finally, artificial intelligence machine learning modeling and analysis are conducted. The results show that the four-step strategy of the system is an effective tool to distinguish the geographical source of PRR samples. The safety and quality of natural products, including PRR, have long been based on the color, shape, taste and organic composition of the sample.

The three-dimensional fluorescence spectrum combined with the stoichiometric modeling detection method to classify and identify the PRR of different origins and origins, which can effectively distinguish the PRR of different origins and samples, and its basic sources are PL and PV. The correct recognition rate reaches 100%. The established method is simple to pre-process the sample, and the detection speed is fast. It can quickly determine whether the PRR sample belongs to the genuine origin, and can effectively eliminate the phenomenon of superiority. It is beneficial for maintaining the market order of traditional Chinese medicinal materials and enhancing the overall quality.At the same time, this method can also provide new ideas and methods for tracing the origins of other traditional Chinese medicinal materials, offering broad application prospects.
